# Westchester County Association

**Published:** 1899-08

**Authors:** 


### SOCIETY PROCEEDINGS.

### ANNUAL DINNER OF THE WESTCHESTER COUNTY VETERINARY MEDICAL ASSOCIATION.

THIS Association held its first annual dinner at Morello's, in West Twenty-ninth Street, New York City. Dr. M. J. Tewey, of Irvington, the President of the Society, occupied the chair, and Prof. James L. Robertson, of the American Veterinary College, was toastmaster. Addresses were made by Prof. Coates, of the American Veterinary College; Assemblyman J. K. Apgar, of Peekskill; Dr. E. F Bush, ex-Mayor of Mt. Vernon; William H. Ely, of Tarrytown; ex-Senator Charles P. McClelland, of Dobb's Ferry; Dr. Richard Buckley, Surgeon for the New York Street Cleaning Department, and others.

Dr. Brush's address was particularly interesting, owing to its startling statements regarding the impure milk sold in the city. "Of the 20,000 babies who die in this great city annually," said he, "at least 35 per cent. die from milk fed to them which is as dangerous as though it contained arsenic or other poisons." He urged the veterinarians present to give more time to the study of the bovine race and less to the horse. The field is open, he said, and it will only be a short time before the source of diphtheria is discovered in the cow, which animal is in many cases the cause of disease in humanity.

